# Supplementary material for: Prompt Framing Modulates Safety in Shoulder and Elbow Red-Flag Vignettes: A Large Language Model Study
Source: Diagnostics (Basel). 2026 May 8;16(10):1439. doi: 10.3390/diagnostics16101439 (PMC13205240; doi:10.3390/diagnostics16101439)
Supplement: Supplementary file 1 [file diagnostics-16-01439-s001.zip › Supplementary Materials/Supplementary Table S1.docx]

**Supplementary Table S1.** Structured comparison of selected prior studies relevant to large language model safety and clinical use in musculoskeletal care

| **Study** | **Context** | **Study type / data** | **Main focus** | **Key limitation** | **Relevance to present study** |
| --- | --- | --- | --- | --- | --- |
| Roustan and Bastardot [1], 2025 | General clinical use of LLMs | Narrative review / perspective | Hallucinations and clinician-facing risks | Not an empirical benchmarking study | Highlights that fluent outputs may still be unsafe in medical contexts |
| Asgari et al. [2], 2025 | Medical text summarization | Safety framework study | Clinical safety and hallucination assessment | Not focused on orthopedic scenarios or triage | Supports safety-oriented evaluation beyond simple correctness |
| Zhou et al. [3], 2025 | Biomedicine and healthcare | Broad review | Opportunities, limitations, and implementation risks of LLMs in healthcare | Broad scope; not musculoskeletal-specific | Provides updated background on deployment, trust, and patient-safety concerns |
| Fiedler et al. [4], 2024 | Shoulder and elbow | Exam-style benchmarking study | Subspecialty knowledge performance | Tests knowledge rather than real-world triage safety | Suggests that orthopedic knowledge performance may be limited |
| Megafu et al. [5], 2025 | Rotator cuff evaluation | Guideline-concordance study | Agreement with AAOS recommendations | Focuses on concordance rather than escalation safety | Shows that guideline alignment does not necessarily ensure safe triage or referral behavior |
| Shah et al. [6], 2024 | Clinical medicine | Methodological review | Prompting strategies in clinical applications | Not specific to urgent musculoskeletal care | Supports prompt framing as a clinically relevant methodological variable |
| Maaz et al. [7], 2025 | Healthcare simulation scenarios | Comparative prompting study | Prompt design and model comparison | Not focused on orthopedic red flags or safety-critical under-triage | Reinforces that prompt structure can materially alter model output |
| Gaber et al. [8], 2025 | Clinical decision support workflows | Workflow evaluation study | Evaluation of LLM workflows in triage, referral, and diagnosis | Not orthopedic-specific | Supports assessment of workflow-sensitive deployment behavior rather than isolated accuracy alone |
| Yu et al. [9], 2018 | Healthcare AI | Broad conceptual review | Clinical opportunities and risks of AI in healthcare | Predates the current LLM ecosystem | Provides foundational clinical AI context for deployment and safety discussion |
| Durán and Jongsma [10], 2021 | Medical AI ethics and trust | Conceptual ethics / epistemology paper | Trust and black-box concerns in medical AI | Not specific to LLMs or orthopedics | Supports the governance and trust dimension of patient-facing deployment |
| Daher et al. [11], 2023 | Shoulder and elbow | Clinical benchmarking study | Diagnosis and management quality | Limited emphasis on safety failure modes | Suggests apparent clinical utility but does not directly evaluate urgent-care safety |
| Tharakan et al. [12], 2024 | Total shoulder and elbow arthroplasty | Patient information study | Quality of answers to common patient questions | Focuses on education rather than triage safety | Relevant to patient-facing deployment but not red-flag escalation |
| Johnson et al. [13], 2025 | Shoulder arthroplasty | FAQ / patient education study | Adequacy of patient-oriented responses | Does not address time-sensitive or red-flag presentations | Shows that acceptable patient education content may still omit important nuance |
| Yong et al. [14], 2024 | Patient communication / complaint handling | Web-based cross-sectional study | Response quality in patient complaint resolution | Not orthopedic-specific | Highlights variability in patient-facing communication quality |
| Espinal et al. [15], 2025 | Lateral epicondylitis | Comparative patient information study | Ability to answer common patient questions | Non-urgent topic; not safety-oriented | Demonstrates that informational adequacy does not equal urgent-care safety |
| Rajkomar et al. [16], 2019 | Machine learning in medicine | Broad review | Clinical use of machine learning in medicine | Not specific to LLMs or musculoskeletal care | Provides general background for interpreting AI-supported clinical reasoning |
| Truhn et al. [17], 2023 | Orthopedic MRI-based recommendations | Pilot study | Treatment recommendation quality | Small pilot; not triage-focused | Suggests that plausible orthopedic recommendations may remain context-limited |
| Hager et al. [18], 2024 | Clinical decision-making | Limitation / mitigation study | Failure modes and mitigation strategies in clinical decision-making | Broad clinical scope, not orthopedic-specific | Supports the need for safeguards and mitigation strategies in clinical deployment |
| Fares et al. [19], 2025 | Shoulder and elbow | Chatbot benchmarking study | Performance on shoulder and elbow questions | Focuses on question-answering rather than red-flag escalation | Supports the distinction between apparent competency and safe triage behavior |
| Topol [20], 2019 | Human-AI clinical integration | Perspective / conceptual review | Convergence of human and artificial intelligence in medicine | Predates contemporary LLM-specific deployment questions | Supports the framing of AI as adjunctive rather than autonomous in clinical care |
| Present study | Shoulder and elbow red-flag scenarios | Standardized fictional vignette study | Safety-critical under-triage, prompt framing, and decision instability | Single model, fictional vignettes, limited red-flag subgroup size | Directly evaluates prompt-sensitive safety behavior in time-sensitive orthopedic presentations |

This table summarizes selected prior studies relevant to large language model performance, safety, prompting, workflow behavior, and clinical deployment, and situates the present study within the existing orthopedic and broader clinical literature.
